# Insuffisance rénale révélant un myélome multiple avec des lésions radiologiques historiques

**DOI:** 10.11604/pamj.2018.31.171.17154

**Published:** 2018-11-12

**Authors:** Nadia Bouchemla, Abderrahim Nadri, Meriem Chettati, Wafaa Fadili, Inass Laouad

**Affiliations:** 1 Service de Nephrologie Hémodialyse Transplantation Rénale, CHU Mohamed VI de Marrakech, Marrakech, Maroc

**Keywords:** Myélome multiple, insuffisance rénale sévère, atteinte osseuse, Multiple myeloma, severe renal failure, bone involvement

## Abstract

The study involved a 54-year old female patient admitted with severe renal failure. She had a 1-month history of progressive stage II dyspnoea associated with chest pain, bone pain and anuria. Clinical examination showed hypertension (160/80mmHg), glomerular disease (urinary protein excretion 2+, blood 2+ and diuresis 300 cc). Pleuropulmonary examination showed diffuse bilateral lower-chest crackling sounds. Laboratory tests objectified severe renal failure with creatinine level 107mg, urea 1.65g/l, hyperkalaemia 7.8 mmol/l, CRP value 78mg/l, normochromic normocytic anemia with hemoglobin concentration 5.7g/dl and leukocytosis 13570 without thrombocytopenia, hyperprotidemia 144g/l, normal serum albumin concentration 33g/l, hypercalcemia 116mg/l and hyperphosphataemia 120mg/l. Serum protein electrophoresis showed monoclonal gamma globulin peak 60 g/l. Immunoelectrophoresis of plasma proteins showed IgG kappa gammapathy. Bence-Jones protein urine test was negative. Myelogram showed plasmocytosis 10%. Profile skull x-rays objectified multiple pre-existing geodes. The patient underwent CDT1 protocol with dexamethazone thalidomide 100 mg and oral endoxan.

## Image en médecine

Il s'agit d'une patiente âgée de 54 ans, admise pour insuffisance rénale sévère. Elle a présenté une dyspnée stade II évoluant depuis un mois avec des douleurs thoraciques et douleurs osseuses associées à une anurie. L'examen clinique a retrouvé une hypertension à 160/80mmHg, un syndrome glomérulaire avec 2+ de protéine, 2+ de sang et une diurèse à 300cc. L'examen pleuro-pulmonaire a montré des rales crépitants basithoraciques bilatéraux. Sur le plan biologique, on a mis en évidence une insuffisance rénale sévère à 107mg de créatinine, urée à 1.65g/l,une hyperkaliémie à 7.8mmol/l, CRP à 78mg/l, une anémie normochrome normocytaire avec une Hb à 5.7g/dl et une hyperleucocytose à 13570 sans thrombopénie. Elle avait une hyperprotidémie à 144g/l, une normoalbuminémie à 33g/l, une hypercalcémie à 116g/l et une hyperphosphorémie à 120mg/l. L'électrophorèse des protéines sériques a objectivé un pic monoclonal de gammaglobulines à 60g/l avec à l'immuno-électrophorèse des protéines plasmatiques une gammapathie de type IgG kappa. La recherche de la protéine de Bence Jones était négative. Le myélogramme a montré une plasmocytose à 10%. Sur le plan radiologique, de multiples géodes à l'emporte pièce ont été visualisé sur la radiographie du crane de profil réalisant un aspect historique. La patiente a été mise sous protocole CDT1 à base de dexaméthazone, thalidomide 100mg et endoxan orale.

**Figure 1:**
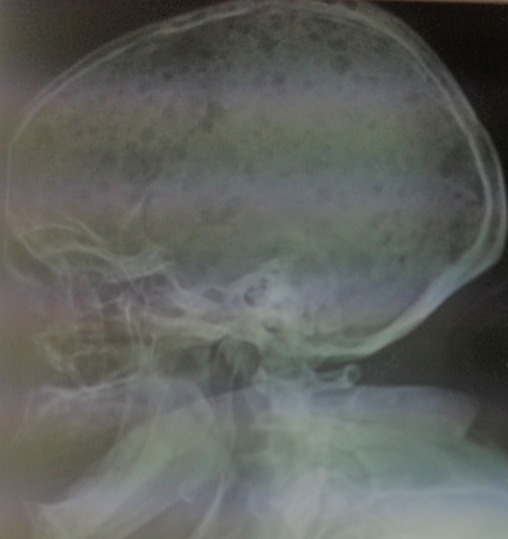
Géodes multiples réalisant une atteinte osseuse historique dans le cadre du myélome multiple

